# Mechanisms of Action of Novel Drugs Targeting Angiogenesis-Promoting Matrix Metalloproteinases

**DOI:** 10.3389/fimmu.2019.01278

**Published:** 2019-06-04

**Authors:** Gregg B. Fields

**Affiliations:** ^1^Department of Chemistry and Biochemistry, Florida Atlantic University, Jupiter, FL, United States; ^2^Department of Chemistry, The Scripps Research Institute/Scripps Florida, Jupiter, FL, United States

**Keywords:** protease inhibitor, clinical trial, metalloproteinase, cancer, angiogenesis, antibody, exosite

## Abstract

Angiogenesis is facilitated by the proteolytic activities of members of the matrix metalloproteinase (MMP) family. More specifically, MMP-9 and MT1-MMP directly regulate angiogenesis, while several studies indicate a role for MMP-2 as well. The correlation of MMP activity to tumor angiogenesis has instigated numerous drug development programs. However, broad-based and Zn^2+^-chelating MMP inhibitors have fared poorly in the clinic. Selective MMP inhibition by antibodies, biologicals, and small molecules has utilized unique modes of action, such as (a) binding to protease secondary binding sites (exosites), (b) allosterically blocking the protease active site, or (c) preventing proMMP activation. Clinical trials have been undertaken with several of these inhibitors, while others are in advanced pre-clinical stages. The mechanistically non-traditional MMP inhibitors offer treatment strategies for tumor angiogenesis that avoid the off-target toxicities and lack of specificity that plagued Zn^2+^-chelating inhibitors.

## Introduction

**Figure 1:**
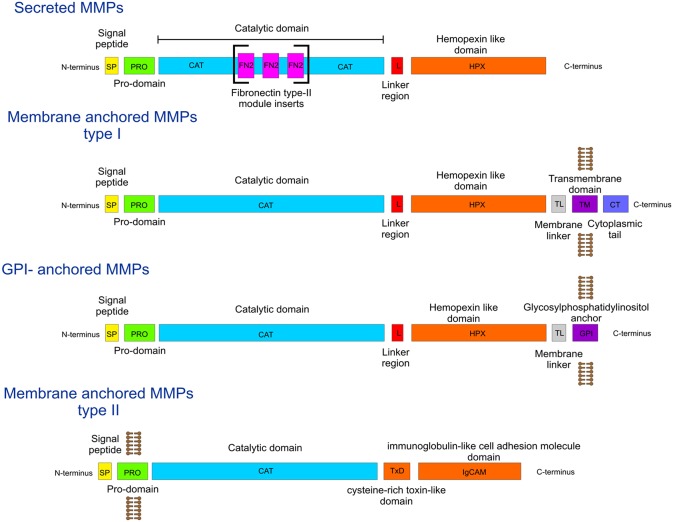
Diagrammatic representation of MMP domain organization.

During the process of angiogenesis (the development of new blood vessels), the extracellular matrix (ECM) is degraded by matrix metalloproteinases (MMPs), facilitating endothelial cell invasion and leading to sprouting of new vessels (1–3). The MMP family (Figure 1) has fairly conserved sequences between species, indicating that they are part of essential biological processes. The domain organization of MMPs is also fairly conserved, as all contain a signal peptide, a pro-domain, and a catalytic (CAT) domain with a Zn^2+^ binding His-Glu-X-X-His-X-X-Gly-X-X-His motif (Figure 1). Most MMPs contain a linker region and a hemopexin-like (HPX) domain (Figure 1). In addition, some harbor specific features such as a furin activation domain (MMP-14/MT1-MMP, MMP-15/MT2-MMP, MMP-16/MT3-MMP, MMP-21, MMP-24/MT5-MMP, MMP-23, and MMP-28), fibronectin type II middle inserts (MMP-2 and MMP-9), and/or a transmembrane domain (MMP-14/MT1-MMP, MMP-15/MT2-MMP, MMP-16/MT3-MMP, and MMP-24/MT5-MMP) (Figure 1).

MMP-9 and MT1-MMP directly regulate angiogenesis, while some studies indicate a role for MMP-2 as well (1, 4). Tumor angiogenesis and growth is reduced in MMP-2 knockout mice (1). MMP-9 has been well-documented as a key contributor to the “angiogenic switch” in cancer progression (5–8). The roles of MMP-9 in angiogenesis include the release of vascular endothelial growth factor (VEGF) and/or basic fibroblast growth factor (FGF-2) (5, 7). Tumor-associated macrophages, once polarized into the M2 phenotype, release VEGF and MMP-9 (9). MT1-MMP contributes to blood vessel invasion, FGF-2-induced corneal angiogenesis, endothelial cell migration and tubulogenesis in three-dimensional collagen matrices, and vascular lumen formation (10–15).

Inhibiting enzymes involved in tumor-driven angiogenesis has been recognized as a potential anticancer therapy (16). Broad spectrum and moderately selective MMP inhibitors have been recognized as possessing antiangiogenic activity (17–19). The majority of MMP inhibitors contain a hydroxamic acid group which chelates the active site Zn^2+^ (20–24). Problems with hydroxamic acid-based metalloprotease inhibitors include the tendency of hydroxamic acids to chelate zinc in a non-selective fashion (25). An often observed side effect of hydroxamic acid-based MMP inhibitors has been musculoskeletal syndrome (MSS). MSS has been attributed to combined inhibition of MMP-1 and a disintegrin and metalloproteinase 17 (ADAM17) (26). A pyrimidine-2,4,6-trione derivative that selectively inhibits MT1-MMP, MMP-2, and MMP-9 is not associated with MSS (27). Recent advances in the development of selective MMP inhibitors have included unique modes of action for inhibiting MMPs implicated in angiogenesis (MMP-2, MMP-9, and MT1-MMP).

## MMP-2/MMP-9 Inhibitors

**Figure 2:**
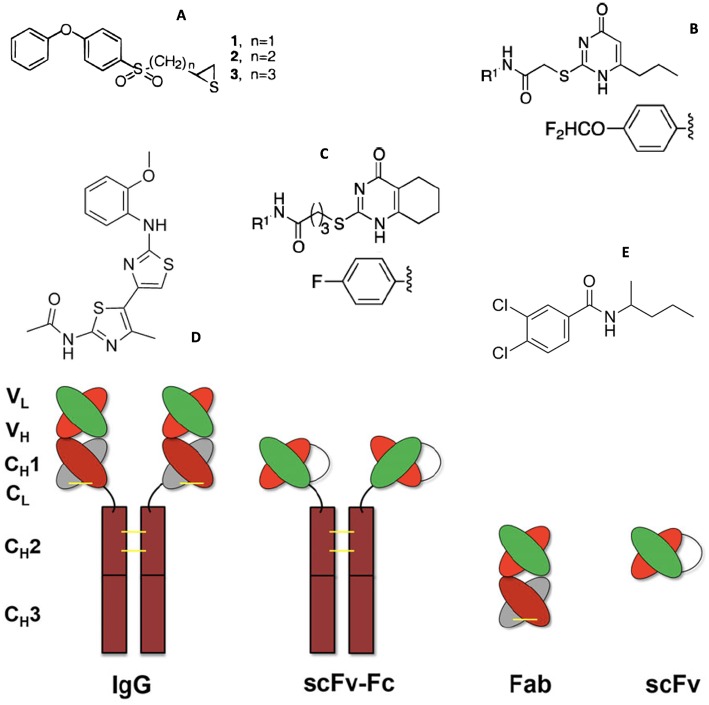
Structures of MMP small molecule inhibitors **(A)** thiiranes (where n = 1 for SB-3CT), **(B)** *N*-[4-(difluoromethoxy)phenyl]-2-[(4-oxo-6-propyl-1 *H*-pyrimidin-2-yl)sulfanyl]-acetamide, **(C)** *N*-(4-fluorophenyl)-4-(4-oxo-3,4,5,6,7,8-hexahydroquinazolin-2-ylthio)butanamide, **(D)** JNJ0966 [*N*-(2-((2-methoxyphenyl) amino)-4′-methyl-[4,5′-bithiazol]-2′-yl)acetamide], and **(E)** NSC405020 [3,4-dichloro-*N*-(1-methylbutyl)benzamide], and (bottom) MMP inhibitory antibodies (IgG) and antibody fragments. Illustrations reprinted with permission from Brown et al. (28), Alford et al. (29), and Santamaria and de Groot (30). Copyright 2000 and 2017 American Chemical Society and 2018 John Wiley and Sons.

Mechanism-based inhibitors selective for MMP-2 and MMP-9 were developed based on the thiirane moiety (Figure 2A) (28). Although it was initially proposed that the thiirane would be activated via coordination with the active site Zn^2+^, allowing for covalent modification by an active site nucleophile (28), subsequent studies revealed a mechanism by which deprotonation at the methylene adjacent to the sulfone occurred, initiating ring opening of the thiirane and formation of a stable Zn^2+^-thiolate complex (31). The thiirane-based inhibitor SB-3CT (Figure 2A) exhibited antiangiogenic and antimetastatic behaviors (32, 33). *In vivo*, SB-3CT was found to be metabolized by several routes, including *p*-hydroxylation, hydroxylation at the methylene adjacent to the sulfone leading to sulfinic acid formation, and glutathione-based Cys conjugation of the thiirane ring (34). α-Methyl variants of SB-3CT had improved metabolic profiles, as only oxidation of the thiirane sulfur was observed (35). Unfortunately, SB-3CT was poorly water soluble. Thiirane-based inhibitors with improved water solubility were subsequently developed (36). ND-322 (which was selective for MMP-2 and MT1-MMP) was found to have antimetastatic activity (37), while the *O*-phosphate prodrug form of SB-3CT crossed the blood-brain barrier (38).

Targeting antibodies (Abs) (Figure 2, bottom) directly to the Zn^2+^ complex in the MMP active site (designated metallobodies) could have superior properties over classical Abs by mimicking the molecular recognition offered by the endogenous inhibitors of MMPs, tissue inhibitor of metalloproteinases (TIMPs), while providing better selectivity (39). Mice were immunized with synthetic organic ligands bound to a metal ion (Zinc-Tripod), which mimicked the MMP catalytic Zn^2+^ complex. This was followed by immunization with the full-length MMP. The immunization procedure yielded function blocking metallobodies (SDS3 and SDS4) directed at the catalytic Zn^2+^ and enzyme surface epitopes in activated MMP-9 (39). Metallobodies SDS3 and SDS4 bound and inhibited MMP-9 with K_D_ = 200 and 20 nM, respectively, and K_i_ = 1 μM and 54 nM, respectively. SDS3 and SDS4 also effectively inhibited MMP-2, but had no inhibitory activity toward MMP-1, MMP-7, MMP-12, or ADAM17, and more than an order of magnitude lower activity toward MT1-MMP. SDS3 was shown, in both prophylactic and therapeutic applications, to protect mice from dextran sodium sulfate-induced colitis (39).

In general, metalloproteinases use the nucleophilic attack of a water molecule as one of the steps of amide bond hydrolysis (40). Water addition to the amide carbonyl results in a tetrahedral transition state. Phosphinic peptides [Ψ{PO_2_H-CH_2_}] are analogs of this transition state and behave as inhibitors of MMPs (41). Phosphinate triple-helical (collagen mimic) MMP inhibitors allow incorporation of specificity elements for both the S and S' subsites of the enzyme. Although binding to the non-primed region of the active site is generally weaker than the primed site to prevent product inhibition (40), it does add sequence diversity and potential selectivity. Triple-helical structure allows for interaction with both the active site and secondary binding sites (exosites) of collagenolytic MMPs (42–44), which include MMP-2, MMP-9, and MT1-MMP (45).

Our laboratory produced a series of triple-helical peptide inhibitors (THPIs) based on GlyΨ{PO_2_H-CH_2_}Leu, GlyΨ{PO_2_H-CH_2_}Val, and GlyΨ{PO_2_H-CH_2_}Ile transition state analogs (42, 46–51). The α1(V)GlyΨ{PO_2_H-CH_2_}Val THPI [C_6_-(Gly-Pro-Hyp)_4_-Gly-Pro-Pro-GlyΨ{PO_2_H-CH_2_}(*R*,*S*)Val- Val-Gly-Glu-Gln-Gly-Glu-Gln-Gly-Pro-Pro-(Gly-Pro-Hyp)_4_-NH_2_], based on the cleavage site in type V collagen by MMP-9 (52), was a selective inhibitor for MMP-2 and MMP-9 (46). The thermal stability of the α1(V)GlyΨ{PO_2_H-CH_2_}Val THPI was greatly reduced compared to the parent substrate (46, 53). We synthesized a stabilized version of the α1(V)GlyΨ{PO_2_H-CH_2_}Val THPI, designated α1(V)GlyΨ{PO_2_H-CH_2_}Val [mep_14, 32_,Flp_15, 33_] THPI, where mep was (2*S*,4*R*)-4-methylproline and Flp was (2*S*,4*R*)-4-fluoroproline (51). α1(V)GlyΨ{PO_2_H-CH_2_}Val [mep_14, 32_,Flp_15, 33_] THPI had a melting point (*T*_m_ value) 18 °C higher than α1(V)GlyΨ{PO_2_H-CH_2_}Val THPI (51). α1(V)GlyΨ{PO_2_H-CH_2_}Val [mep_14, 32_,Flp_15, 33_] THPI exhibited K_i_ values against MMP-2 and MMP-9 of 189.1 and 90.6 nM, respectively, at 25°C, and 2.24 and 0.98 nM, respectively, at 37°C (51).

Triple-helical peptides (THPs) have been found to be reasonably stable to general proteolysis, as observed *in vitro* in mouse, rat, and human serum and/or plasma and *in vivo* in rats (54–58). The stability of THPs has allowed for their administration orally (59). The α1(V)GlyΨ{PO_2_H-CH_2_}Val [mep_14, 32_,Flp_15, 33_] THPI was effective *in vivo* in a mouse model of multiple sclerosis, reducing clinical severity and weight loss (51).

## MMP-2 Selective Inhibitors

Chlorotoxin (ClTx) is 36-residue peptide isolated from the venom of the Israeli Yellow scorpion *Leiurus quinquestriatus* (60). ClTx preferentially binds neuroectodermal tumors and exhibits antiangiogenic and anti-invasion activity (61–65). ClTx selectively inhibits MMP-2 in a dose-dependent manner (K_D_ ~ 115 nM) (62). The ClTx interaction with a membrane complex of chloride channel-3 (ClC-3) and MMP-2 (66) has been used to create numerous cancer imaging agents (63, 65, 67–69). ClTx can pass through the blood-brain barrier (65), and has yielded promising preclinical and clinical results in the treatment of glioblastoma (64, 68).

## MMP-9 Selective Inhibitors

Mouse mAb REGA-3G12, a selective inhibitor of MMP-9, was prepared using MMP-9 as antigen (70). REGA-3G12 recognized the MMP-9 Trp116 to Lys214 region, located in the CAT domain but not part of the Zn^2+^ binding site (71). REGA-3G12 bound to MMP-9 with K_D_ = 2.1 nM (70). REGA-3G12 prevented interleukin-8-induced mobilization of hematopoietic progenitor cells in rhesus monkeys (72). A single chain variable fragment (scFv) (Figure 2, bottom) derived from REGA-3G12 selectively inhibited MMP-9 compared to MMP-2 (73). Gelatin hydrolysis was inhibited 44% at a scFv concentration of 5 μM (73).

Two monoclonal anti-MMP-9 antibodies, AB0041 and AB0046, were shown to inhibit tumor growth and metastasis in a surgical orthotopic xenograft model of colorectal carcinoma (74). AB0046 improved immune responses to tumors, as the inhibition of MMP-9 reversed MMP-9 inactivation of T-cell chemoattractant CXCR3 ligands (CXCL9, CXCL10, and CXCL11) (75). A humanized version of AB0041, GS-5745 (Andecaliximab), was generated for use in clinical trials (74). GS-5745 was found to bind to MMP-9 near the junction between the pro-domain and CAT domain, distal to the active site, and (a) inhibited proMMP-9 activation and (b) non-competitively inhibited MMP-9 activity (76). GS-5745 bound to MMP-9 with ~150-400-fold weaker affinity compared with proMMP-9 (K_D_ = 2.0–6.6 vs. 0.008–0.043 nM) (76). GS-5745/Andecaliximab has been evaluated under several clinical trials. A randomized placebo controlled phase 1b single and multiple ascending dose-ranging clinical trial on 72 patients diagnosed with moderately to severely active ulcerative colitis (UC) showed that GS-5745 was safe, well-tolerated, and could be used as a potential therapeutic agent for UC (77). A phase 2/3 UC study with 165 patients treated over 8 weeks further indicated that GS-5745 was well-tolerated (78). A phase 1b trial investigating the safety, pharmacokinetics, and disease-related outcomes for 15 rheumatoid arthritis patients (ClinicalTrials.gov Identifier NCT02176876) demonstrated that GS-5745 was safe, with adverse events that were only grade 1 or 2 in severity and no indication of MSS (79).

Several non-active site small molecule MMP-9 inhibitors have been described. *N*-[4-(difluoromethoxy)phenyl]-2-[(4-oxo-6-propyl-1*H*-pyrimidin-2-yl)sulfanyl]-acetamide (Figure 2B) bound selectively to the MMP-9 HPX domain with K_D_ = 2.1 μM and inhibited tumor growth and lung metastasis in MDA-MB-435 mouse models (80). Based on this lead compound a library of analogs was generated, and *N*-(4-fluorophenyl)-4-(4-oxo-3,4,5,6,7,8-hexahydroquinazolin-2-ylthio)butanamide (Figure 2C) emerged as a more potent inhibitor (K_D_ = 320 nM) (29). This compound prevented association of proMMP-9 with the α4β1 integrin and CD44, resulting in the dissociation of epidermal growth factor receptor (EGFR) from the β1 integrin subunit and CD44 (29). High-throughput screening led to the identification of compound JNJ0966 [*N*-(2-((2-methoxyphenyl)amino)-4′-methyl-[4,5′-bithiazol]-2′-yl)acetamide] (Figure 2D), which bound selectively to proMMP-9 with K_D_ = 5.0 μM (81). JNJ0966 inhibited the activation of proMMP-9 and the migration of HT1080 cells, and was able to penetrate the blood-brain barrier (81).

## MT1-MMP Selective Inhibitors

Several selective MT1-MMP inhibitory antibodies and antibody fragments have been described (27, 30, 82–84). Screening a human Fab display phage library resulted in the development of DX-2400, a selective, fully human MT1-MMP inhibitory antibody (K_i_ = 0.8 nM) (27, 85). DX-2400 was a competitive inhibitor of MT1-MMP (85). DX-2400 inhibited tumor MT1-MMP activity, resulting in the inhibition of MDA-MB-231 primary tumor growth but not MCF-7 tumor growth in xenograft mouse models (85). DX-2400 also inhibited metastasis (85), and enhanced tumor response to radiation therapy (86).

Recombinant human scFv antibodies (Figure 2, bottom) were generated against the MT1-MMP HPX domain (87). Two scFv antibodies, CHA and CHL (K_D_ = 10.7 and 169 nM, respectively), were found to have differing activities. CHL inhibited MT1-MMP binding to collagen, while CHA had the opposite effect, yet both scFv antibodies inhibited HT1080 invasion of type I collagen. CHA inhibited CD44 shedding and endothelial cell sprouting from endothelial cell/fibroblast co-cultures in type I collagen, while CHL had no effect on either activity (87).

Monoclonal antibody (mAb) 9E8 (K_D_ = 0.6 nM) inhibited MT1-MMP activation of proMMP-2, but not other MT1-MMP catalytic activities (88). mAb 9E8 bound to the Pro163 to Gln174 loop in the MT1-MMP CAT domain (89). This loop region is present in the CAT domain of MT1-MT6-MMPs, but is not found in all other MMPs. mAb 9E8 prevented formation of the MT1-MMP•TIMP-2•proMMP-2 complex required for proMMP-2 activation by interfering with TIMP-2 binding (89). Another antibody raised against the loop region, LOOP_Ab_, also inhibited MT1-MMP activation of proMMP-2, but not MT1-MMP collagenolysis (90).

The LEM-2/15 antibody was generated using a cyclic peptide mimicking the MT1-MMP CAT domain V-B loop (residues 218-233) (91). A minimized Fab fragment (Figure 2, bottom) of LEM-2/15 was designed, and possessed a reasonable binding affinity compared to the intact antibody (K_D_ = 2.3 vs. 0.4 nM, respectively) (92). The Fab fragment was a non-competitive inhibitor of MT1-MMP activities, including collagenolysis (92). The Fab fragment of LEM-2/15 induced a conformational change in MT1-MMP by destabilizing the exposed region of the V-B loop, ultimately narrowing the substrate binding cleft (30, 84, 92). Treatment with the Fab fragment of LEM-2/15 significantly increased the ability of virally infected mice to fight off secondary *Strep. pneumoniae* bacterial infection (93). Treatment with the Fab fragment of LEM-2/15, before or after infection, helped to maintain tissue integrity (93).

Human scFv-Fc (Figure 2, bottom) antibody E3 bound to the MT1-MMP CAT domain and inhibited type I collagen binding (94). A second generation E3 clone (E2_C6, K_D_ = 0.11 nM) inhibited tumor growth and metastasis (94).

Human antibody Fab libraries were synthesized where the Peptide G sequence (Phe-Ser-Ile-Ala-His-Glu) (95) was incorporated into complementarity determining region (CDR)-H3 (96). Fab 1F8 exhibited EC_50_ = 8.3 nM against the MT1-MMP CAT domain, and inhibited MT1-MMP CAT domain activity with K_i_ = 110 nM (96).

Screening of a phage displayed synthetic humanized Fab library led to the identification of Fab 3369 (97). Fab 3369 inhibited the activity of the MT1-MMP CAT domain with IC_50_ = 62 nM (97). IgG 3369 treatment of MDA-MB-231 mammary orthotopic xenograft mice reduced lung metastases, collagen processing, and tumor density of CD31^+^ blood vessels (97).

It has been noted that antibody antigen binding sites are not complimentary to the concave shape of catalytic clefts, as antigen binding sites are planar or concave (84). To overcome this, the convex-shaped paratope of camelid antibodies was incorporated into the human antibody scaffold (98). Fab 3A2 bound selectively to MT1-MMP CAT domain outside of the active site cavity with K_D_ = 4.8 nM, and was a competitive inhibitor with K_i_ = 9.7 nM (98, 99). Fab 3A2 inhibited MT1-MMP collagenolysis and reduced metastasis in a melanoma mouse model (99).

Virtual ligand screening of the NCI/NIH Developmental Therapeutics Program ~275,000 compound library resulted in the identification of compound NSC405020 [3,4-dichloro-*N*-(1-methylbutyl)benzamide] (Figure 2E), a small molecule MT1-MMP HPX domain inhibitor (100). NSC405020 inhibited MT1-MMP homodimerization but not proMMP-2 activation or catalytic activity toward a peptide substrate. NSC405020 reduced the collagenolytic activity of MCF7-β3/MT1-MMP cells and was effective *in vivo*, as intratumoral injections reduced tumor size significantly (100).

## Critical Overview

Tumor growth is limited without the ability of the tumor to create its own blood supply (101). The use of antiangiogenic therapeutic agents is viewed as beneficial due to (a) the prevention of new blood vessel formation and/or (b) the normalization of tumor-associated vasculature (102). Normalizing the tumor-associated vasculature can enhance the penetration of therapeutic agents (102, 103). Clinically utilized antiangiogenic agents typically target VEGF or the VEGF receptor (VEGFR), or are multikinase inhibitors (102). Significant improvement in overall survival and prolonged progression-free survival was observed when angiogenesis inhibitors were applied in gastric cancer (104). Anti-VEGFR-2 and multikinase inhibitor treatments were more efficacious than anti-VEGF treatment (104). This was suggested to be due to blocking only VEGF-A in the latter treatment (104). Thus, angiogenesis targeting via MMP inhibition could be very efficacious based on the potential broader impact than just VEGF-A inhibition (as discussed in the Introduction). The ability of the combination of angiogenesis inhibition and chemotherapy to prolong progression-free survival in patients with gastric cancer was dependent upon the antiangiogenic agent used (104).

Antiangiogenic therapies can have serious side effects, such as bleeding, venous or arterial thromboembolisms, proteinuria, and hypertension, and can also increase drug resistance, cancer invasion, and metastasis (102, 104–106). An obvious concern is that antiangiogenic approaches can negatively impact capillaries and blood flow in healthy tissues (104). Additionally, targeting VEGF can lead to upregulation of other pro-angiogenic factors (107, 108). All in all, side effects from the use of angiogenesis inhibitors are often viewed as manageable (104, 105, 109).

Unique modes of action have been used to develop antibody-based, triple-helical peptide, and small molecule inhibitors of MMPs implicated in angiogenesis. The selective, small molecule MMP-9 and MT1-MMP inhibitors do not yet have preferred affinities, but represent a promising start based on their novel mechanisms of inhibition. Clinical trials utilizing antibodies have provided evidence that selective MMP inhibitors do not induce MSS. Unfortunately, antibodies are subject to proteolysis, may be removed from circulation rapidly, and are costly. Nonetheless, antibodies have provided truly selective, high affinity MMP inhibitors. Selective, high affinity inhibitors can be developed for MMPs based on triple-helical structure. THPIs have excellent pharmacokinetic properties compared with other peptide-based therapeutics. The mechanistically non-traditional MMP inhibitors offer treatment strategies for tumor angiogenesis that avoid the off-target toxicities and lack of specificity that plagued Zn^2+^-chelating inhibitors.

One must consider that when applied as antiangiogenic agents, MMP inhibitors may have the undesired effect of (a) limiting turnover of already existing tumor vessels and (b) disrupting vascular homoeostatis, where normal vessel turnover and other related activities are needed. This would be dependent upon which MMP was targeted. For example, MT1-MMP has been shown to contribute to both angiogenesis and vascular regression in an aortic ring model (110). Inhibition of MT1-MMP catalytic activity following the vessel growth phase resulted in reduced vascular regression due to inhibition of collagenolysis (110). Vessels are destabilized by MT1-MMP shedding of Tie-2 from endothelial cells (111), and thus enzyme inhibition could stabilize tumor vessels (103). In similar fashion, TIMP-2 and TIMP-3 were found to stabilize newly formed vascular networks by (a) inhibiting regression and (b) preventing further endothelial cell tube morphogenesis (112). The action of TIMP-2 and TIMP-3 was correlated to MT1-MMP activity, and thus inhibition of MT1-MMP could stabilize vascular networks (112). Deletion of MT1-MMP or inhibition of MT1-MMP activity resulted in increased vascular leakage (103). In this latter case, MT1-MMP was proposed to modulate TGFβ availability, with decreased TGFβ levels impacting vascular homoeostatsis (103). MT1-MMP shedding of endoglin (CD105) results in the release of sEndoglin, which inhibits angiogenesis (113). MMP-9 contributes to edema prevention, which is a component of vascular homoeostasis (103). MMP-2 cleavage of ECM biomolecules leads to disruption of endothelial cell β1 integrin binding and subsequent signaling (114, 115). In turn, disruption of signaling leads to a decrease in MT1-MMP production (114).

Another consideration for MMP inhibition is the effect on the production of antiangiogenic agents, such as angiostatin (from plasminogen), endostatin (from type XVIII collagen), arresten (from the α1(IV) collagen chain), canstatin (from the α2(IV) collagen chain), and tumstatin (from the α3(IV) collagen chain). MMP-9 is capable of generating angiostatin (116, 117), endostatin (118, 119), arresten (120), canstatin (120), and tumstatin (120, 121). However, the redundancy of proteases capable of generating these agents (116, 118, 120) suggests that inhibiting one (such as MMP-9) may have little effect on these particular antiangiogenic activities.

While selective MMP inhibitors are greatly needed, often overlooked is that the timing of MMP inhibitor application is also critical (see above). Application of a broad spectrum MMP inhibitor (marimostat) in combination with gemcitabine significantly improved survival in pancreatic cancer patients with disease confined to the pancreas (122). Presurgical treatment with an oral MMP inhibitor improved survival from 67 to 92% in a mouse breast cancer model (123). As discussed earlier, MMP-9 is a key contributor to the angiogenic switch during carcinogenesis of pancreatic islets (5). However, MMP-9 deficiency in pancreatic ductal adenocarcinoma (PDAC) mouse models resulted in more invasive tumors and an increase in desmoplastic stroma (124). The absence of MMP-9 led to increased interleukin 6 levels in the bone marrow, which activated tumor cell STAT3 signaling and promoted PDAC invasion and metastasis (124). Thus, MMP-9 represents an anti-target in the later stage of pancreatic cancer. The “window of opportunity” for MMP inhibitor application is often in premetastatic disease (125).

## Author Contributions

The author confirms being the sole contributor of this work and has approved it for publication.

### Conflict of Interest Statement

The author declares that the research was conducted in the absence of any commercial or financial relationships that could be construed as a potential conflict of interest.

## Abbreviations

Abs: antibodies
ADAM: a disintegrin and metalloproteinase
CAT: catalytic
ClC-3: chloride channel-3
ClTx: chlorotoxin
ECM: extracellular matrix
EGFR: epidermal growth factor receptor
FGF-2: basic fibroblast growth factor 2
Flp, (2*S*: 4*R*)-4-fluoroproline
HPX: hemopexin-like
mep, (2*S*: 4*R*)-4-methylproline
MMP: matrix metalloproteinase
MSS: musculoskeletal syndrome
PDAC: pancreatic ductal adenocarcinoma
scFv: single chain variable fragment
THP: triple-helical peptide
THPI: triple-helical peptide inhibitor
TIMP: tissue inhibitor of metalloproteinase
UC: ulcerative colitis
VEGF: vascular endothelial growth factor.
